# Biosynthesis of Silver Nanoparticles Using Seasonal Samples of Sonoran Desert Propolis: Evaluation of Its Antibacterial Activity against Clinical Isolates of Multi-Drug Resistant Bacteria

**DOI:** 10.3390/pharmaceutics14091853

**Published:** 2022-09-02

**Authors:** Pablo Mendez-Pfeiffer, Manuel G. Ballesteros-Monrreal, Jesus Gaona-Ochoa, Josue Juarez, Marisol Gastelum-Cabrera, Beatriz Montaño-Leyva, Margarita Arenas-Hernández, Liliana Caporal-Hernandez, Jesús Ortega-García, Edwin Barrios-Villa, Carlos Velazquez, Dora Valencia

**Affiliations:** 1 Department of Chemistry-Biology and Agropecuary Sciences, Universidad de Sonora, H. Caborca, Hermosillo 83600, Sonora, Mexico; 2 Departamento de Física, Universidad de Sonora, Hermosillo 83000, Sonora, Mexico; 3 Departamento de Investigación y Posgrado en Alimentos, Universidad de Sonora, Hermosillo 83000, Sonora, Mexico; 4 Posgrado en Microbiología, Centro de Investigación en Ciencias Microbiológicas, Instituto de Ciencias, Benemérita Universidad Autónoma de Puebla, Ciudad Universitaria, Puebla 72570, Pue, Mexico; 5 Department of Chemistry-Biology, Universidad de Sonora, Hermosillo 83000, Sonora, Mexico

**Keywords:** Sonoran Desert propolis, silver nanoparticles, propolis nanoparticles, multi-drug resistant bacteria

## Abstract

Multi-drug resistant (MDR) bacteria have gained importance as a health problem worldwide, and novel antibacterial agents are needed to combat them. Silver nanoparticles (AgNPs) have been studied as a potent antimicrobial agent, capable of countering MDR bacteria; nevertheless, their conventional synthesis methods can produce cytotoxicity and environmental hazards. Biosynthesis of silver nanoparticles has emerged as an alternative to reduce the cytotoxic and environmental problems derived from their chemical synthesis, using natural products as a reducing and stabilizing agent. Sonoran Desert propolis (SP) is a poplar-type propolis rich in polyphenolic compounds with remarkable biological activities, such as being antioxidant, antiproliferative, and antimicrobial, and is a suitable candidate for synthesis of AgNPs. In this study, we synthesized AgNPs using SP methanolic extract (SP-AgNPs) and evaluated the reduction capacity of their seasonal samples and main chemical constituents. Their cytotoxicity against mammalian cell lines and antibacterial activity against multi-drug resistant bacteria were assessed. Quercetin and galangin showed the best-reduction capacity for synthesizing AgNPs, as well as the seasonal sample from winter (SPw-AgNPs). The SPw-AgNPs had a mean size of around 16.5 ± 5.3 nm, were stable in different culture media, and the presence of propolis constituents was confirmed by FT-IR and HPLC assays. The SPw-AgNPs were non-cytotoxic to ARPE-19 and HeLa cell lines and presented remarkable antibacterial and antibiofilm activity against multi-drug resistant clinical isolates, with *E. coli* 34 and ATCC 25922 being the most susceptible (MBC = 25 μg/mL), followed by *E. coli* 2, 29, 37 and PNG (MBC = 50 μg/mL), and finally *E. coli* 37 and *S. aureus* ATCC 25923 (MBC = 100 μg/mL). These results demonstrated the efficacy of SP as a reducing and stabilizing agent for synthesis of AgNPs and their capacity as an antibacterial agent.

## 1. Introduction

Antibiotic resistance is an important challenge today, since it is considered one of the greatest threats to human health, affecting developed and developing countries equally. Multi-drug resistant (MDR) bacteria are pathogens with resistance to three or more classes of antibiotics; however, pathogens resistant to all clinically available antibiotics have been reported. Due to this problem, the World Health Organization (WHO) predicts that by 2050, infections caused by MDR bacteria will be the leading cause of mortality worldwide [1,2].

To reduce the effects of MDR bacteria, alternatives to conventional drugs are being investigated. A reliable alternative is the use of nanomaterials, in particular, silver nanoparticles (AgNPs), which have a strong antimicrobial activity and have been used as antiseptic and antimicrobial agents [3]. However, one of the problems presented by the conventional synthesis of AgNPs is the production of toxic residues derived from the reducing and stabilizing agents that are used in their manufacture, so an alternative approach to their synthesis called “biosynthesis” or “green synthesis” is being attempted, to replace the commonly used reducing and stabilizing agents [4]. It has been reported that some plant extracts such as *Pinus nigra* [5], microorganisms [6], and even bacterial DNA [7] can be used in the synthesis of AgNPs.

Propolis is a chemically complex resin produced by bees (*Apis mellifera*); its chemical composition varies depending on the available plant sources and external factors such as weather and seasonality [8,9,10]. Propolis collected from arid regions such as the Sonoran Desert in Mexico contain a wide variety of flavonoids and polyphenolic compounds and is considered a poplar-type propolis with a broad array of biological activities, such as being immunomodulatory [11], antiproliferative [12,13], antimicrobial [14], and antioxidant [15]. Therefore, Sonoran Desert propolis (SP) could be used as a reducing and stabilizing agent to synthesize silver nanoparticles with antimicrobial potential.

In this work, we synthesized AgNPs using SP extract (SP-AgNPs), describing the differences in the reducing process by chemical differences in the seasonal samples of SP, as well as their main chemical constituents. In addition, we evaluated the antimicrobial and antibiofilm activities of SP-AgNPs against clinical isolates of MDR bacteria, to determine their efficacy as a potential therapeutic alternative.

## 2. Materials and Methods

### 2.1. Chemical Reagents

Silver nitrate (≥99%), sodium hydroxide (NaOH), bovine serum albumin (BSA), HPLC grade methanol, ethanol, formic acid, fetal bovine serum, Mueller–Hinton, Luria–Bertani, and Dulbecco’s Modified Eagle media, as well as L-asparagine (98%), L-arginine monohydrochloride (≥98%), L-glutamine solution (200 mM), sodium pyruvate solution (100 mM), and penicillin-streptomycin solution (1000 U/1 U per mL) were purchased from Sigma-Aldrich Co. (St. Louis, MO, USA). Pinocembrin (≥95%), pinobanksin-3-O-acetate (≥95%) (Pb-3-O-Ac), chrysin (≥95%), galangin (≥95%), and quercetin (≥95%) were purified from propolis collected in Ures, Sonora, Mexico (N 2927.1810, W 110 23.398) by chromatographic isolation procedures over silica gel 60 (0.015–0.040 mm; Merck KGaA), using progressive proportions of ethyl acetate in hexane as the mobile phase (Tedia Company, Fairfield, OH, USA). Caffeic acid phenethyl ester (CAPE) was synthesized according to the esterification of caffeic acid and phenethyl alcohol, based on the procedure described by Grunberger et al. [16].

### 2.2. Propolis Samples and Extract Preparation

Seasonal samples of Sonoran Desert propolis were collected from the winter 2014 to autumn 2015 from twelve hives located in the “Tecolote” farm (N 31_02.180, W 112_02.580) in the state of Sonora, between the municipalities of Caborca and Altar. For the extraction, 10 g of propolis were mixed with 50 mL of methanol for three days and filtered. Next, methanol was evaporated under reduced pressure, to obtain the propolis methanolic extract. Samples were stored at −20 °C [15].

### 2.3. Synthesis of Silver Nanoparticles

AgNPs were synthesized following the reduction of silver ions using propolis methanolic extract, the seasonal sample from spring was used for the standardization of the synthesis conditions, varying the concentration of AgNO_3_ (0.5–1.0 mM), concentration of propolis (50–100 µg/mL), temperature (25 °C and 70 °C), and reaction time (1 h and 2 h).

Stock solutions of propolis samples were prepared in DMSO (40 mg/mL). For the synthesis of AgNPs, 5 mL of Milli-Q water were mixed with different concentrations of propolis extract (50, 70, and 100 μg/mL) under magnetic stirring, and the pH of the solution was adjusted to 10.1 with NaOH (1 M). Next, AgNO_3_ was added, to obtain a final concentration of 0.5 mM or 1 mM. Experiments were performed at 25 and 70 °C and 1 and 2 h of reaction. Upon standardization of the best synthesis conditions, seasonal samples of Sonoran Desert propolis extract (spring, autumn, winter, and summer), as well as some of their chemical constituents (pinocembrin, pinobanksin-3-O-acetate, galangin, chrysin, quercetin, and CAPE) were used for the synthesis of AgNPs. AgNPs samples were centrifuged at 12,000× g for 30 min and washed with Milli-Q water for further characterization.

### 2.4. Characterization of Silver Nanoparticles

The formation of Sonoran Desert propolis silver nanoparticles (SP-AgNPs) was verified by the presence of the localized surface plasmon resonance (LSPR) band with a wavelength scan from 250 to 700 nm using a UV-Vis spectrophotometer Thermo Scientific Evolution 60 S (Thermo Fischer Scientific, Waltham, MA, USA). Hydrodynamic diameter and zeta potential were determined using a Zetasizer NanoZs (Malvern Instruments Ltd., Malvern, Worcestershire, UK). The morphology was analyzed by atomic force microscopy (AFM) using a Jeol JSPM-4210 (Jeol, Peabody, MA, USA). Transmission electron microscopy (TEM) images were obtained using a Transmission electron microscope (TEM JEOL JEM1011 (Jeol, Peabody, MA, USA). The images were analyzed using WSxM 5.0 software (Julio Gómez Herrero & José María Gómez Rodríguez) and imageJ 1.53s software (National Institutes of Health, Bethesda, MD, USA) [17].

The polyphenolic profile of seasonal samples of SP-AgNPs was determined by HPLC-UV-DAD, using an Agilent 1290 Infinite series with a C18 column (Zorbax Eclipse Plus C18 4.6 × 100 mm 2.5 Micron). The method used was: 1% formic acid in H_2_O (solvent A) and methanol (solvent B) at 1 mL/min at a gradient of: time 0–2 min, A 70%, B 30%; 5 min, A 60%, B 40%; 10 min, A 55%, B 45%; 50 min, A 40%, B 60%; 80 min, A 20%, B 80%, followed by washing and re-equilibrating of the column. The chromatogram was recorded at 280 nm [15].

The presence of the propolis extract functional groups on the winter SP-AgNPs (SPw-AgNPs) was confirmed by FTIR-ATR. The spectra were obtained using a Perkin-Elmer spectrometer (Waltham, MA, USA) at a resolution of 4 cm^−1^, from 4000 to 400 cm^−1^.

Stability assays were performed by measuring the intensity of the LSPR band of SPw-AgNPs in different media (Muller-Hinton broth and Dulbecco’s modified eagle medium supplemented with 5% (D5F) and 10% of fetal bovine serum (D10F)) for a period of 144 h using a UV-Vis spectrophotometer MultiskanGO MicroPlate reader (Thermo Fischer Scientific, Waltham, MA, USA).

### 2.5. Cell Culture and Cytotoxic Effect

Human cervix adenocarcinoma cells (HeLa) and human normal retinal pigment epithelium cells (ARPE-19) were purchased from the American Type Culture Collection (ATCC; Rockville, MD, USA). Cells were cultured in DMEM media supplemented with 5% of FBS, L-asparagine (98%), L-arginine monohydrochloride (≥98%), and L-glutamine solution (200 mM), as well as sodium pyruvate solution (100 mM) and penicillin-streptomycin solution (1000 U/1 U per mL) (D5F).

The cytotoxic effect of SPw-AgNPs was assessed using an MTT assay [18,19]. Initially, 50 µL of cell suspension was seeded into each well in a 96-well flat-bottom plate and incubated for 24 h at 37 °C and 5% of CO_2_. Next, 50 µL of SPw-AgNPs and SPw extract were dispersed in D5F media and placed into each well at the desired concentration (100–12.5 µg/mL), and the plate was incubated for 48 h at 37 °C and 5% of CO_2_. Four hours prior to the incubation time, media was removed, the cells were washed with PBS, and 100 µL of fresh D5F media was added to each well. Next, 10 µL of MTT (5 mg/mL) was added to each well, and the plate was incubated for 4 h at 37 °C and 5% CO_2_. Next, 100 µL of acidic isopropanol was added to each well, to solubilize the formazan crystals, and the absorbance was measured using a MultiskanGO MicroPlate reader (Thermo Fischer Scientific, Waltham, MA, USA) at 570 nm and a reference wavelength of 630 nm.

### 2.6. Antibacterial Activity

Antibacterial activity of SPw-AgNPs was evaluated following the broth microdilution method of the Clinical and Laboratory Standards Institute (CLSI) for the determination of minimum inhibitory concentration (MIC) and minimum bactericidal concentration (MBC) [20]. The microorganisms used for this study were *Escherichia coli* ATCC 25922, *Staphylococcus aureus* ATCC 25923, and clinical isolates of multi-drug resistant *Escherichia coli* (strains 2, 27, 29, 34, 37, and PNG), previously characterized [21,22]. Briefly, SPw-AgNPs were resuspended in Mueller–Hinton media and diluted to different concentrations (100–12.5 µg/mL); next, 100 µL of nanoparticles were placed in 96-well plates (Costar, Corning). The bacterial inoculum was adjusted from a fresh culture to the 0.5 McFarland standard (1 × 10^8^ CFU/mL), then the inoculum was diluted at 1:20, and 10 µL of bacteria were placed in each well. Plates were incubated at 37 °C for 23 h and read every hour in a MultiskanGO MicroPlate reader (Thermo Fischer Scientific, Waltham, MA, USA) at 620 nm. To determine MBC, 10 µL of each well was inoculated on a Mueller–Hinton agar plate. The SPw-AgNPs concentration at which no bacterial growth was observed in the culture medium was considered the MBC.

### 2.7. Anti-Biofilm Assay

Biofilm formation was carried out following a previously reported method with some modifications [23]. Strains of multi-drug resistant *E. coli* (strains 2, 27, 29, 34, 37, PNG, and ATCC 25922) and *S. aureus* ATCC 25923 were plated on Luria–Bertani (LB) agar and incubated at 37 °C overnight. Then, 5 mL of LB broth was inoculated with one CFU and incubated at 37 °C overnight. Next, 300 µL of a 1:14 dilution from each sample was added into a 96-U-well Polyvinyl chloride (PVC) microplate and incubated for 48 h at 37 °C. Next, the planktonic cells were removed, and the microplate was washed three times with sterile PBS. Next, 150 µL of TBS media containing different concentrations of SPw-AgNPs (200–12.5 µg/mL) was added to each well, and the microplate was incubated at 37 °C for 24 h. The supernatant was eliminated, and the microplate was rinsed with sterile PBS. Twenty µL of 0.1% crystal violet solution was added and incubated at room temperature for 15 min. The microplate was then vigorously washed with distilled water, 230 µL of absolute ethanol was added to each well, and the plate was incubated at room temperature for 2 min. OD was read at 600 nm with a MultiskanGO MicroPlate reader (Thermo Fischer Scientific, Waltham, MA, USA). An average of three separated reads for each strain was obtained and interpolated against a calibration curve of BSA (400, 200, 100, and 50 mg/mL).

### 2.8. Statistical Analyses

The results were analyzed using Two Way ANOVA Tukey’s multiple comparisons test, using GraphPad Prism 6.04 software for Windows (GraphPad Software, La Jolla, CA, USA, www.graphpad.com, accessed on 5 March 2022). The level of significance was considered a *p*-value ≤ 0.05.

## 3. Results

### 3.1. Synthesis and Characterization of Silver Nanoparticles

**Figure 1:**
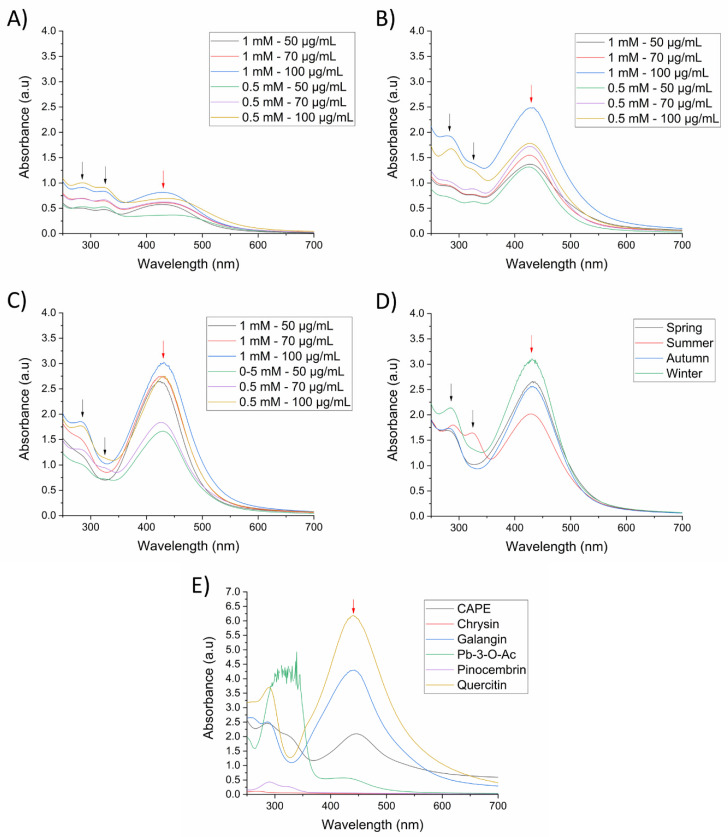
UV-Vis spectra of SP-AgNPs at different synthesis conditions (AgNO_3_—Propolis extract). (**A**) 25 °C and 1 h reaction time; (**B**) 70 °C and on 1 h reaction time; (**C**) 70 °C and 2 h reaction time; (**D**) seasonal samples of SP-AgNPs; (**E**) AgNPs synthesized with propolis chemical constituents. The concentration of seasonal samples of SP and all chemical constituents used for the synthesis of AgNPs was 100 µg/mL, except for quercetin (20 µg/mL). Synthesis was carried out with 1 mM AgNO_3_ at 70 °C for 2 h. Black arrows (spectral bands at 280 and 330 nm of SP); red arrows (LSPR of silver nanoparticles). Pb-3-O-Ac: Pinobanksin-3-O-acetate. Spectra are representative of three independent experiments.

Silver nanoparticles were initially prepared and standardized using a spring propolis sample at different synthesis conditions, and nanoparticle formation was monitored by identification of its LSPR by UV-Vis spectrophotometry, from a range of 250–700 nm. First, we evaluated the reduction capacity of SP (50–100 µg/mL) at room temperature (25 °C) with 0.5 mM or 1 mM of silver precursor (AgNO_3_) and 1 h of reaction time at constant stirring and pH 10.1. In Figure 1A, we can observe the presence of the LSPR at 430 nm, demonstrating the formation of AgNPs. Nevertheless, the intensity of absorption is indicative of a low nanoparticle concentration. In contrast, upon increasing the temperature to 70 °C, more AgNPs were formed, especially at 1 mM of AgNO_3_ and 100 µg/mL of propolis, as observed by the absorption of the LSPR (Figure 1B). To ensure the total reduction of silver by the propolis extract, we increased the reaction time to 2 h (Figure 1C), where the intensity of the LSPR increased in comparison to 1 h of reaction, indicating major formation of AgNPs. Absorption bands around 280 nm and 330 nm can be observed and are related to the SP extract (black arrows).

Upon establishing the best synthesis conditions for AgNPs (1 mM AgNO_3_, 100 µg/mL propolis extract, 70 °C, and 2 h reaction time), we wanted to evaluate the differences in nanoparticle formation using previously characterized seasonal samples of propolis extract [15]. In Figure 1D, based on the intensity of the band corresponding to the LSPR, we can observe that the winter propolis sample presented a higher reductive capacity, followed by the spring, autumn, and summer. These results indicated that silver reduction and nanoparticle formation was affected by quantitative differences of compounds in seasonal propolis samples [15].

**Figure 2:**
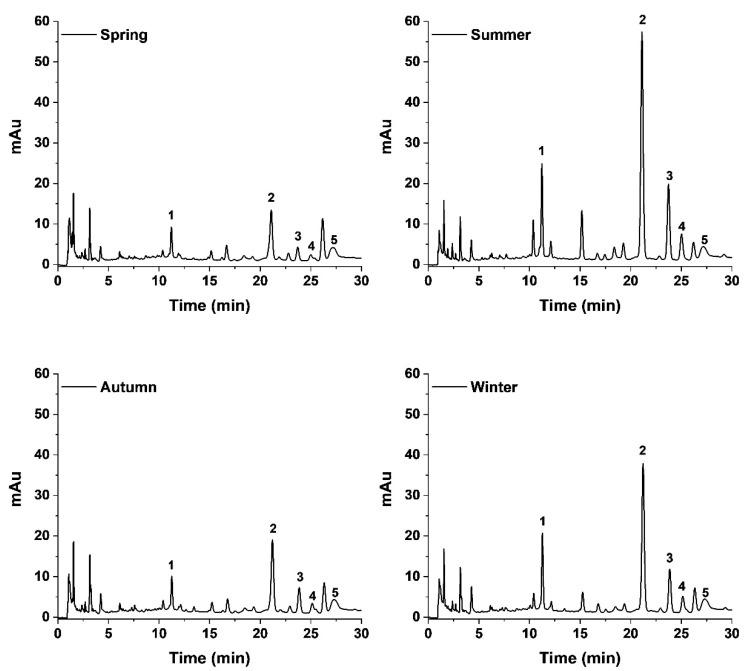
Polyphenolic profile of seasonal samples of SP-AgNPs. Five main constituents of Sonoran propolis were identified: quercetin (1), pinocembrin (2), pinobanksin-3-O-acetate (3), chrysin (4), and galangin (5). mAu: milli-absorbance units.

To further understand the role of the propolis chemical constituents in the synthesis of AgNPs, we evaluated the main chemical markers of the propolis samples (pinocembrin, pinobanksin 3-O-acetate, galangin, chrysin and quercetin), as well as CAPE, which has previously been reported in Sonoran Desert propolis as a highly active molecule [19]. There was no formation of AgNPs using pinocembrin and chrysin as reducing agents, while CAPE and Pb-3-O-Ac produced AgNPs with low absorption of the LSPR, demonstrating its poor reduction capacity (Figure 1E); it is noteworthy that the spectrum of Pb-3-O-Ac was above the detection limits of the equipment, due to its high concentration, nevertheless the LSPR of the formed AgNPs was too low. On the other hand, galangin and quercetin demonstrated a high reducing power, as observed by the high intensity of the LSPR, especially quercetin, which required a lower concentration for AgNPs synthesis (20 µg/mL) compared to the other constituents (100 µg/mL). Furthermore, changes in the polyphenolic profile of seasonal samples of propolis upon the synthesis of the AgNPs were determined using HPLC-UV-DAD. We can observe in Figure 2 that the chromatogram of the supernatant of the four seasonal samples of SP-AgNPs are similar to those previously reported for these SP seasonal samples [15]; we identified the presence of quercetin (1), as well as the presence of the four compounds previously reported (pinocembrin [2], Pb-3-O-Ac [3], chrysin [4], and galangin [5]). A proportion of the main components of SP were retained upon the synthesis of AgNPs when we compared this to the previously reported profile of SP.

We characterized the seasonal samples of propolis silver nanoparticles (SP-AgNPs) by size, zeta potential, and morphology. The hydrodynamic diameter obtained by DLS shows differences in size only in SP-AgNPs synthesized with the winter propolis sample (68.0 ± 1.7 nm), meanwhile, the size for autumn (60.2 ± 1.1 nm), spring (59.9 ± 0.4 nm), and summer (58.9 ± 0.2 nm) was the same. The zeta potential values ranged from −31.6 ± 1.1 to −52.0 ± 1.5 mV, with the winter samples having with a higher value (Table 1).

**Figure 3:**
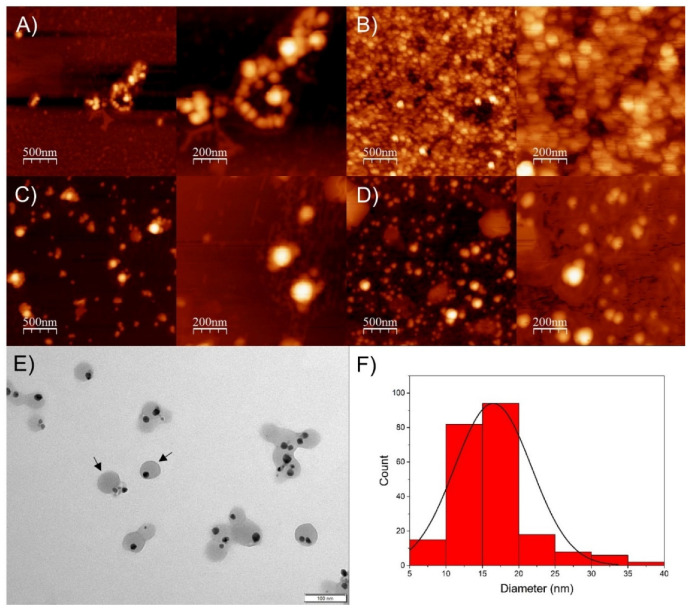
Atomic force microscopy and transmission electron microscopy of seasonal samples of SP-AgNPs. (**A**) Spring SP-AgNPs; (**B**) Summer SP-AgNPs; (**C**) Autumn SP-AgNPs; (**D**) Winter SP-AgNPs; (**E**) TEM image of winter SP-AgNPs, and (**F**) histogram of winter SP-AgNPs from TEM images. Black arrows: SP-AgNPs surrounded by propolis extract.

As observed using AFM images (Figure 3A–D), the SP-AgNPs presented a spherical shape, with size ranging from 50 to 100 nm, similar to the results obtained by DLS.

Based on these results, we selected SP-AgNPs synthesized with winter propolis extract (SPw-AgNPs) for transmission electron microscopy (TEM), FT-IR characterization, stability assay, and evaluation of its biological activity. As shown by TEM images (Figure 3E), the nucleus of the SPw-AgNP was surrounded by the propolis extract (black arrows), and the mean diameter of the nanoparticles was 16.5 ± 5.3 nm, smaller than the diameter determined by DLS and AFM.

**Figure 4:**
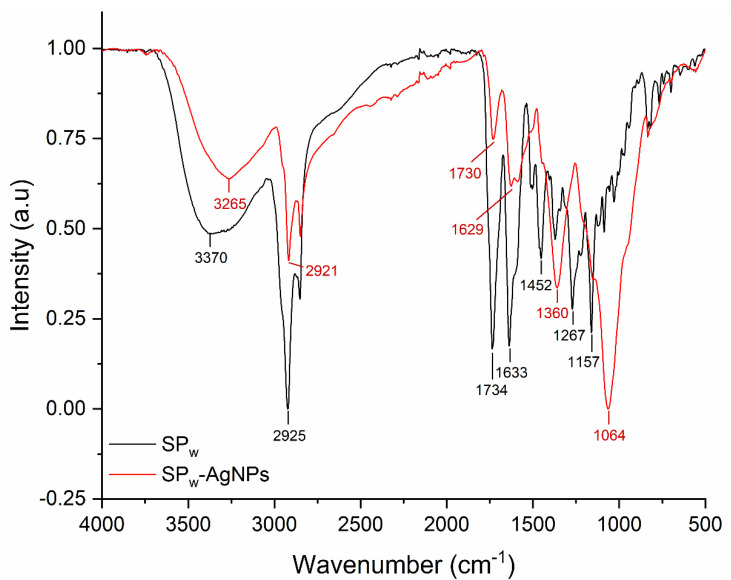
FT-IR spectra of SPw (black line) and SPw-AgNPs (red line).

The FT-IR of SPw-AgNPs and winter SP extract (SPw) is shown in Figure 4. The major peaks in SPw-AgNPs were observed at 3265, 2921, 1629, 1360, and 1064 cm^−1^; meanwhile, for SP, the major peaks were located at 3370, 2925, 1734, 1633, 1452, 1267, and 1157 cm^−1^. For instance, the signals between 3265 and 3370 cm^−1^ correspond to the stretching vibration of -OH groups, and the signal around 2925 and 2921 cm^−1^ corresponds to C-H stretching vibration; meanwhile, the signals around 1360, 1054, and 1157 cm^−1^ could be from heterocyclic compounds (C-O-C).

The stability of SPw-AgNPs was evaluated in different media, such as Muller–Hinton broth, D5F, and D10F, by measuring the intensity and position of the SLPR at different timepoints. We observed a decrease in the SPLR band on the three media, especially at 96 h of incubation. In addition, a band localized at around 560 nm appeared after 144 h of incubation in D5F and 96 h in D10F (S1).

The cytotoxic activity of SPw-AgNPs and SPw was evaluated using an MTT assay against ARPE-19 and HeLa cell lines (S2). As observed for SP-AgNPs, the viability of both cell lines was maintained above 75%, even at the higher concentration evaluated (100 μg/mL), demonstrating to be non-toxic against these cells. Meanwhile, SPw exerted a dose-dependent toxic effect in both cell lines, with the HeLa cell line being the most susceptible, with viability percentages of around 50% (12 μg/mL) and as low as 5%, at the higher concentration evaluated (100 μg/mL).

### 3.2. Antibacterial Activity

To evaluate the antibacterial activity of SPw-AgNPs, we determined the minimum inhibitory concentration, minimum bactericidal concentration, and effect on the bacterial growth at 24 h of SPw-AgNPs against *E. coli* ATCC 25922 and *S. aureus* ATCC 25923 (control strains), as well as clinical isolates of multi-drug resistant uropathogenic *E. coli* with different resistance phenotypes (MDR, XDR, and PDR) (phenotypic and genotypic characteristics of analyzed clinical isolates are shown in the Supplementary Material Table S1). The most susceptible strains against SPw-AgNPs based on their MBC were *E. coli* ATCC 25922 and the clinical isolates *E. coli* 2 and *E. coli* 34, with an MBC of 25 µg/mL, followed by strains of *E. coli* 29, 37, and PNG with an MBC of 50 µg/mL, finally the most resistant strains were *E. coli* 27 and *S. aureus* ATCC 25923 with an MBC of 100 µg/mL. Interestingly, the MIC and MBC value was the same for all strains, except for *E. coli* 2 (MIC = 25 µg/mL and MBC = 50 µg/mL) and *S. aureus* ATCC 25923 (MIC = 25 µg/mL and MBC = 100 µg/mL) were the amount of SPw-AgNPs needed to kill the bacteria was two and four times higher than to inhibit their growth, respectively (Table 2).

**Figure 5:**
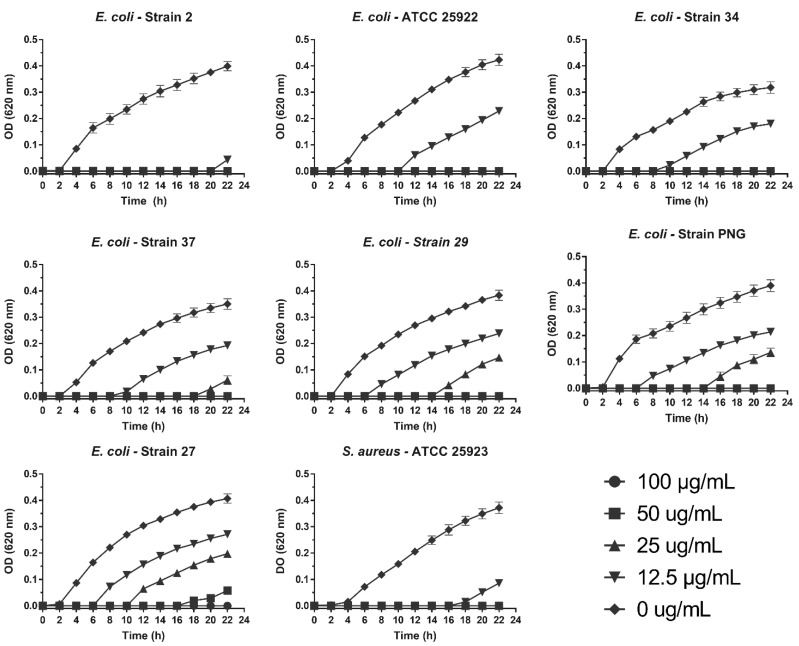
Antibacterial activity of SPw-AgNPs against ATCC and clinical isolates of multi-drug resistant bacteria. Bacteria were tested with different doses of SPw-AgNPs (100–12.5 µg/mL). Data represent the mean of three independent experiments ± standard deviation.

We determined the effect of the treatments on the growth curve of the analyzed clinical isolates. Interestingly, a delay of 6 to 21 h was observed in the microbial growth curve of all pathogens evaluated at concentrations lower than the MIC. This was most noticeable in *E. coli* strain 2, with a 21-h delay at 12.5 µg/mL (Figure 5).

### 3.3. Antibiofilm Activity

**Figure 6:**
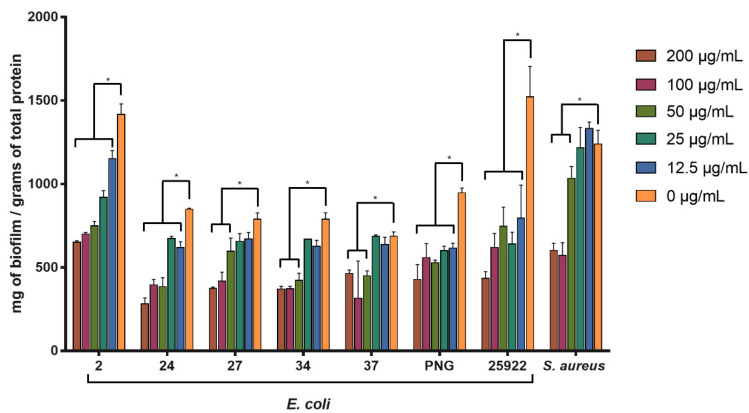
Antibiofilm activity of SPw-AgNPs against ATCC and clinical isolates of multi-drug resistant bacteria. Bacterial biofilms were tested with different doses of SPw-AgNPs (200–12.5 µg/mL). Data represent the mean of three independent experiments ± standard deviation. * Statistical significance between treated and untreated biofilm (*p* < 0.05).

The activity of SPw-AgNPs against pre-formed biofilm was evaluated for all our clinical isolates and ATCC models at 24 h of treatment (200–12.5 μg/mL). SPw-AgNPs were capable of significantly reducing the biomass of biofilms at the lowest tested concentration (12.5 μg/mL) for the strains of *E. coli* 2, 24, PNG, and ATCC 25922; nevertheless, higher concentrations of SPw-AgNPs were needed to observe significant differences in the reductions of the biofilm biomass for strains *E. coli* 27, 34, 37, and *S. aureus* ATCC 25923, compared to the untreated control (Figure 6). 

## 4. Discussion

It has been reported that, depending on their characteristics, silver nanoparticles have a potent bactericidal effect on bacterial pathogens of medical importance, such as *S. aureus* and *E. coli* [24,25,26]. However, one of the main problems associated with using these nanosystems is their cytotoxic effect on human cell lineages, a characteristic mainly associated with their synthesis method. Due to this, the search for stabilizing agents that reduce the unwanted effects of silver nanoparticles, while maintaining or enhancing their bactericidal effect, is of great interest. Previous studies have described Sonoran Desert propolis as poplar-type propolis with a wide variety of biological activities, including antimicrobial activity [14,27]. In addition, seasonal samples of SP collected from the region of Caborca, Sonora, Mexico, have been described as potent antioxidants [15,19,27], suggesting that they could have a good reducing capacity for the synthesis of AgNPs. We proposed SP extracts as a reducing agent for the synthesis of silver nanoparticles and combined their antimicrobial activity for the development of a nanosystem against multi-drug resistant bacteria.

We previously reported quantitative differences in the chemical composition of seasonal propolis samples, affecting their biological activities, such as the antioxidant and antiproliferative activity [13,15]. In these samples, four main constituents were identified (pinocembrin, pinobanksin-3-O-acetate, chrysin, and galangin), observing quantitative differences, depending on the season of collection, which could affect its silver reducing capacity. We observed that SP collected in summer presented a lower reducing capacity for synthesis of AgNPs, despite having the highest concentration of pinocembrin and pinobanksin-3-O-acetate (Pb-3-O-Ac). Meanwhile, propolis collected in winter demonstrated the highest reducing capacity and formation of AgNPs. These differences could be attributed to changes in the concentrations of minor chemical constituents with higher reductive power, such as galangin, which is in higher concentration in winter samples, and other non-identified compounds, and not by the most abundant compounds such as pinocembrin and Pb-3-O-Ac [15].

To assess the role of these chemical constituents in the synthesis of AgNPs, we used the main chemical compounds identified in the samples, as well as CAPE and quercetin, which were reported in other samples of SP with high biological activities. Pinocembrin, pb-3-O-Ac, and chrysin did not form AgNPs, demonstrating that these constituents do not contribute to the silver reduction process, which is in accordance with the low antioxidant capacity of these molecules [28,29]. Interestingly, pinocembrin and Pb-3-O-Ac are the main constituents of propolis from summer samples and are in higher concentrations compared to the other seasonal samples. Meanwhile, galangin and quercetin induced strong formation of AgNPs; galangin was present in higher concentrations in winter propolis samples compared to the other seasonal samples [15]. Quercetin was also present in all propolis samples, as observed by HPLC, and could be the main chemical constituent responsible for the synthesis of AgNPs, along with other minor molecules with high reducing power that have not been identified. Reports where AgNPs were synthesized using flavonoids have been observed, which are in agreement with our results, where quercetin and galangin presented great reducing capacity [30,31]; these two constituents also presented a high antioxidant activity in FRAP assays, which correlates with their capacity to reduce metallic ions [32].

Since only some components of SP are responsible for the synthesis of AgNPs, we wanted to observe if there were changes in the polyphenolic profile of SP after the synthesis process. The chromatograms showed the presence of the four previously identified polyphenols (pinocembrin, Pb-3-O-Ac, galangin, and chrysin) at the same retention time and proportions previously described for these samples [15]. This suggests that despite not contributing to the reducing process, components such as pinocembrin, pb-3-O-Ac, and chrysin are retained in the nanoparticles and can still contribute to their stabilization and possibly to their antimicrobial activity. The FT-IR spectra of SP and SP-AgNPs from the winter propolis sample showed shared signals, with small differences due to the chemical changes of propolis during the reduction process, demonstrating the presence of organic compounds from propolis in the AgNPs [33,34]. It has been reported that flavonoids, phenolic acids, proteins, terpenoids, and polyphenols are responsible for reducing silver ions to AgNPs [35,36]; nevertheless, it is necessary to determine the constituents responsible for this process in a complex matrix, such as a natural extract, for its standardization.

The nanoparticles’ size, stability, surface charge, and morphology play a crucial role in modulating their interaction with the target, and thereby their biological activity. All our SP-AgNPs presented a hydrodynamic diameter of around 60 nm, which agrees with AgNPs synthesized using other types of propolis extracts, with sizes ranging from 50 to 100 nm [24,37]. Nevertheless, TEM images of SPw-AgNPs showed nanoparticles with an average diameter of 16 nm. DLS and TEM analysis differences are common, since TEM allows us to observe the nanoparticle core and differentiate it from the propolis matrix. A strong negative zeta potential was also observed, which is indicative of the deposition of the propolis extract on the surface of the nanoparticles, and the high zeta potential values above |30| mV demonstrate its colloidal stability through electrostatic repulsion.

SPw-AgNPs presented a low cytotoxic activity against both cell lines tested (HeLa and ARPE-19) compared to SPw. For therapeutic purposes, NPs should not be cytotoxic to mammalian cells. Previous reports demonstrated that AgNPs could be cytotoxic, depending on the stabilizing agents used, and also that propolis alone can present an antiproliferative activity against various cell lines [10,13,19]. Nevertheless, our SP-AgNPs showed no cytotoxic activity against the tested cell lines, compared to SPw. This could be attributed to the oxidation of the constituents of propolis and their arrangement in the nanoparticle surface, changing their possible active sites and reducing their biological activity [38].

Clinical bacterial isolates’ virulence and resistance phenotypes can be very diverse, differ widely from ATCC or control strains, and could play an important role in the susceptibility against new antibacterial compounds; therefore, it is important to routinely evaluate their biological activity against fully characterized bacterial strains. All the strains behaved differently in our study, depending on the SPw-AgNP concentration. Nevertheless, the MIC and MBC for all our strains were lower than 100 μg/mL, demonstrating good antibacterial activity, compared to other similar studies where metallic nanoparticles were synthesized with propolis extract. Barbosa et al. synthesized silver nanoparticles using Brazilian propolis, and determined a MBC value of 16,650 μg/mL for *E. coli* [39]. Botteon et al. synthesized gold nanoparticles using different extracts of Brazilian red propolis, where only the propolis hexane fraction presented an MBC of 50 μg/mL against *E. coli*; meanwhile, the other fractions (crude extract, ethyl acetate and dichloromethane) presented MBC values higher than 198 μg/mL [38].

Interestingly, there was no relationship between the virulence and resistance phenotype of the strains and the susceptibility to the silver nanoparticles. Furthermore, although the growth of all strains remained static at different AgNPs concentrations, it started to increase after several hours of treatment; this could be attributed to a metabolic adaptation of bacteria at sub-inhibitory concentrations of silver nanoparticles [40,41], as well as other phenomena, such as bacterial persistence. Persistent bacteria are described as cells with low metabolic activity, and although their persistence mechanism has not been fully characterized, it has been associated to toxin–antitoxin systems [42,43]. These persistent cells have been reported to be resistant to antibiotics and heavy metals [44].

Different resistance mechanisms against silver ions and silver nanoparticles have been reported. In *E. coli*, the Cus efflux system can release metal ions, such as copper and silver, to the extracellular environment, along with the downregulation of the OMPs porins, reducing the internalization of metal ions. Other resistance mechanisms are present in plasmids, such as the *sil* operon, which encode for Ag+ binding/efflux systems [45]. The differential response of our bacterial strains to SPw-AgNPs could be due to the co-selection of these silver resistance mechanisms, along with their antibiotic resistance characteristics, and this requires further characterization.

Nevertheless, the antibacterial activity of SPw-AgNPs could be attributed, not only to silver, but also to the propolis constituents present in them. It has been reported that propolis flavonoids can cause cell wall damage and inhibition of membrane function, such as potassium loss, along with a reduction of ATP production and decrease of bacterial mobility [46,47]. Numerous chemical constituents responsible for propolis antibacterial activity have also been determined, such as, pinocembrin, naringenin, Pb-3-O-Ac, and galangin [14,48,49].

SPw-AgNPs could reduce the pre-formed biofilm of all our strains. However, higher concentrations of SPw-AgNPs above the MIC (50–200 μg/mL) were needed to observe a considerable reduction of the biofilm matrix, which is in accordance with biofilm’s capacity to resist antibacterial agents.

Based on our results, future studies should include strains with fully characterized virulence and resistance phenotypes, to assess the relevance of new treatments against bacterial infections in the clinical environment.

## 5. Conclusions

The seasonal samples of Sonoran propolis methanolic extract showed a differential capacity to produce silver nanoparticles. The reducing capacity of Sonoran propolis depends on their chemical variations, depending on the season of collection, with the extract from winter being the one with the highest capacity for synthesis of silver nanoparticles. Our study suggests that chemical constituents such as quercetin and galangin highly contribute to the reduction process of silver; meanwhile, other constituents such as pinocembrin, pinobanksin-3-O-acetate, and chrysin had a poor reduction capacity, but continue to be part of the AgNPs. Our SPw-AgNPs exerted remarkable antibacterial and antibiofilm activity compared to other propolis synthesized AgNPs, although this was dependent on the strain. More studies focusing on the evaluation of fully characterized clinical isolates should be performed, to better understand the possible role of AgNPs as a new antibacterial agent.

## Figures and Tables

**Table 1:** Size and zeta potential of seasonal samples of SP-AgNPs.

SP-AgNPs	Size (Hd.nm)	Zeta Potential (mV)
Spring	59.9 ± 0.4	−47.5 ± 3.1
Summer	58.9 ± 0.2	−31.6 ± 1.1
Autumn	60.2 ± 1.1	−32.4 ± 1.3
Winter	68.0 ± 1.7	−52.0 ± 1.5

Hd.nm: Hydrodynamic diameter in nanometers; mV: millivolts. Data represent the mean of three independent experiments ± standard deviation.

**Table 2:** Minimum inhibitory concentration (MIC) and minimum bactericidal concentration (MBC) of bacteria treated with SPw-AgNPs.

Bacteria	MIC (µg/mL)	MBC (µg/mL)
*S. aureus* ATCC 25923	25	100
*E. coli* ATCC 25922	25	25
*E. coli* 2	25	50
*E. coli* 27	100	100
*E. coli* 29	50	50
*E. coli* 34	25	25
*E. coli* 37	50	50
*E. coli* PNG	50	50

MIC and MBC were determined by three independent experiments.

## Data Availability

Not applicable.

## Supplementary Materials

The following supporting information can be downloaded at: https://www.mdpi.com/article/10.3390/pharmaceutics14091853/s1, Figure S1: Stability assay of SPw-AgNPs on different culture media. The absorbance of the LSPR band of SPw-AgNPs was monitored for 144 h, Figure S2: Cytotoxic activity of SPw-AgNPs and SP against ARPE-19 and HeLa cell lines. Data represent the mean of three independent experiments ± standard deviation, Table S1: Phenotypic and genotypic characteristics of analyzed clinical isolates of multidrug resistant *E. coli*.
