# Supplementary material for: Biosynthesis of Silver Nanoparticles Using Seasonal Samples of Sonoran Desert Propolis: Evaluation of Its Antibacterial Activity against Clinical Isolates of Multi-Drug Resistant Bacteria
Source: Pharmaceutics. 2022 Sep 2;14(9):1853. doi: 10.3390/pharmaceutics14091853 (PMC9503092; doi:10.3390/pharmaceutics14091853)
Supplement: Supplementary file 1 [file pharmaceutics-14-01853-s001.zip › Supplementary Figure S1.pdf]

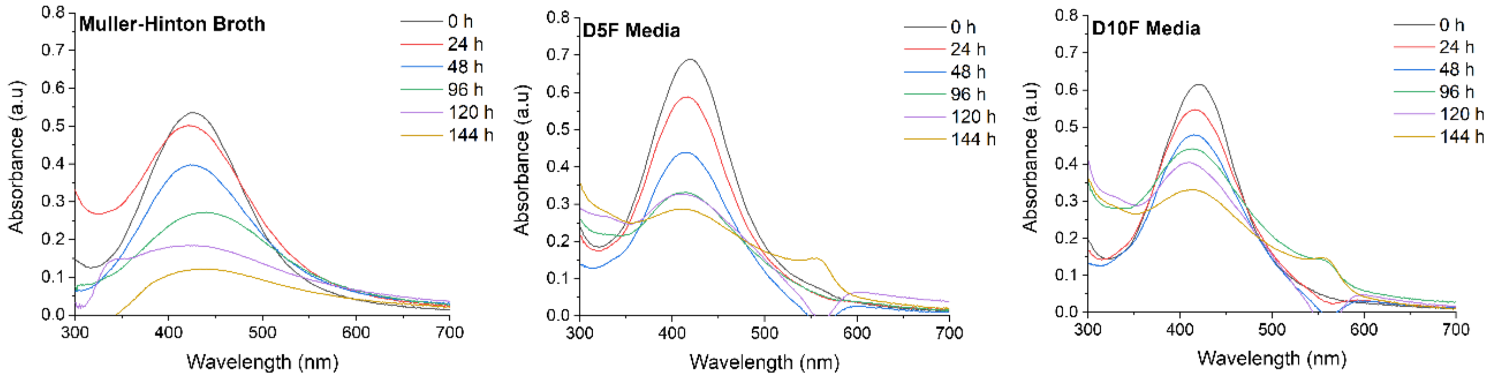

Supplementary Figure S1. Stability assay of SPw-AgNPs on different culture media. The absorbance of the LSPR band of SPw-AgNPs was monitored for 144 h.
