# Supplementary material for: Biosynthesis of Silver Nanoparticles Using Seasonal Samples of Sonoran Desert Propolis: Evaluation of Its Antibacterial Activity against Clinical Isolates of Multi-Drug Resistant Bacteria
Source: Pharmaceutics. 2022 Sep 2;14(9):1853. doi: 10.3390/pharmaceutics14091853 (PMC9503092; doi:10.3390/pharmaceutics14091853)
Supplement: Supplementary file 1 [file pharmaceutics-14-01853-s001.zip › Supplementary Figure S2.pdf]

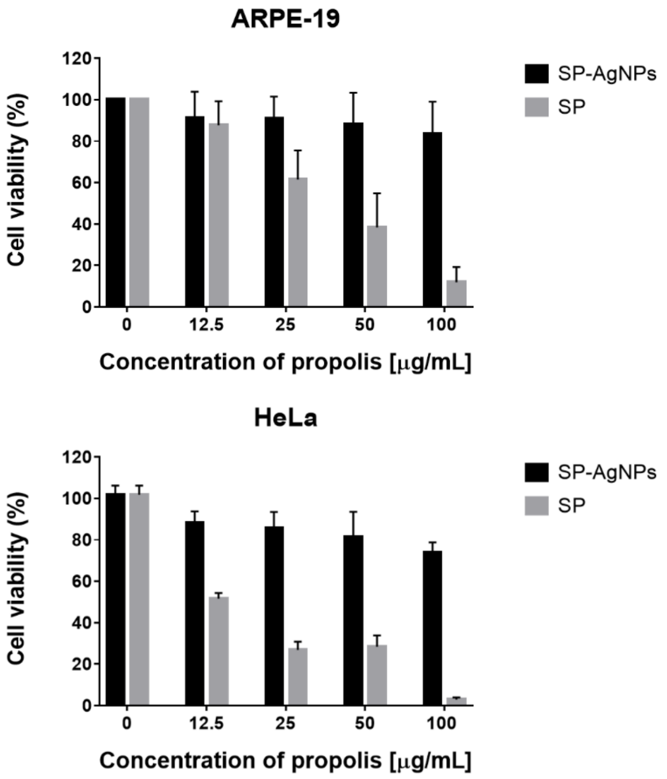

Supplementary Figure S2. Cytotoxic activity of SPw-AgNPs and SP against ARPE-19 and HeLa cell lines. Data represent the mean of three independent experiments ± standard deviation.
