# Supplementary material for: Biosynthesis of Silver Nanoparticles Using Seasonal Samples of Sonoran Desert Propolis: Evaluation of Its Antibacterial Activity against Clinical Isolates of Multi-Drug Resistant Bacteria
Source: Pharmaceutics. 2022 Sep 2;14(9):1853. doi: 10.3390/pharmaceutics14091853 (PMC9503092; doi:10.3390/pharmaceutics14091853)
Supplement: Supplementary file 1 [file pharmaceutics-14-01853-s001.zip › Supplementary Table S1.pdf]

Supplementary Table S1: phenotypic and genotypic characteristics of analyzed clinical isolates of multidrug resistant *E. coli.*

Strain	Virotype	Phenotype	Resistance genotype	Resistotype	MDR classification
***E. coli* 2**	*hlyA, kpsM, vat, cnf-1, traT, iucD, iutA, feoB*	Biofilm, Motility, ESBL+	CTX-M 1 y 8, CTX-M-9. TEM, CTX-M-2, CTX-M-151, qnrB, aac(6')-Ib	GM, CIP, NOR, LVX, TSX, AMP, CFX, CFZ, CTX, CRO, FEP, ATM, AMC, AMS, TE	XDR
***E. coli* 27**	*fimH, sfaD/focC, fliCD, sat, kpsM, traT, iroN, iucD, fyuA, feoB*	Biofilm, Motility, CAR+	CTX-M 1 y 8, CTX-M-2	AMK, GM, CIP, NOR, LVX, TSX, AMP, CX, CFZ, CTX, CRO, AMC, MEM	XDR
***E. coli* 29**	*fimH, sfaD/focC, fliCD, cnf-1, traT, iha, feoB*	Biofilm, Motility, Capsule, ESBL+	CTX-M-2, TEM	AMK, GM, CIP, NOR, LVX, TSX, AMP, CFX, CTX, AMC, TE	MDR
***E. coli* 34**	*fimH, papG-II, fliCD, sat, hlyA, kpsM, vat, cnf-1, traT, iroN, iucD, fyuA, feoB*	Biofilm, Motility, Capsule, Hemolysis, ESBL+	CTX-M-2	AMK, GM, CIP, NOR, LVX, TSX, MAC, AMP, CFX, CFZ, CTX, CRO, AMC, TE	XDR
***E. coli* 37**	*fimH, fliCD, sat, hlyA, kpsM, vat, iroN, iucD, fyuA, iha, feoB*	Biofilm, Motility, Capsule, Hemolysis, ESBL+	TEM, CTX-M-2	TSX, AMP, AMC, AMS	MDR
***E. coli* PNG**	*iucD, sat, fimH, iha, papG, papA, hlyA*	Motility	-	AMK, GM, NET, AN, CIP, OFX, NOR, LVX, TSX, MAC, C, AM, CF, CFX, CFZ, CTX, CRO, FEP, ATM, AMC, FOS, CL, TE, ETP	PDR

fimH: Type 1 pilus adhesin; ***sfaD/focC:*** F1C pilus; ***papG-II:*** Type P pilus Adhesin allele 2; ***fliCD:*** Flagellin subunit/flagellar cap; ***hlyA:*** α-hemolysin;; ***kpsM:*** Capsular variant; ***vat:*** Autotransporter secreted toxin; ***cnf-1:*** Necrotizing cytotoxic factor; ***traT:*** Complement resistance associated protein; ***iucD:*** Aerobactin; ***iutA:*** Aerobactin receptor; ***feoB:*** Ferrous iron transporter B; ***fyuA:*** Ferric yersiniabactin uptake receptor; ***sat:*** Secreted autotransporter toxin; ***iha:*** Enterobactin***; iroN:*** Salmochelin; ***papA:*** Type P pilus; **ESBL+:** Extended spectrum betalactamase phenotype; **CAR+:** Positive carbapenemases phenotype; **CTX-M; TEM;SHV:** β-lactamases associated genes; **qnrB: aac(6')-Ib:** Quinolones resistance associated genes (plasmids). **GM:** Gentamicin; **CIP:** Ciprofloxacin; **NOR:** Norfloxacin; **LVX:** Levofloxacin**; TSX:** Cotrimoxazole; **AMP:** Ampicillin; **CFX:** Cefuroxime**; CTX:** Cefotaxime; **CRO:** Ceftriaxone; **FEP:** Cefepime; **ATM:** Aztreonam; **AMC:** Amoxicillin - Clavulanic acid; **AMS:** Ampicillin-Sulbactam; **TE:** Tetracycline; **AMK:** amikacin; **CF:** Cephalothin; **CL:** Colistin; **ETP:** Ertapenem; **MEM:** Meropenem; **MDR:** Multi-drug resistant; **XDR:** Extensively drug-resistant; **PDR:** Pandrug-resistant.
